# Mycobacterium abscessus Hand Infection Mimicking Dactylitis Due to Spondyloarthritis: A Case Report

**DOI:** 10.7759/cureus.80740

**Published:** 2025-03-17

**Authors:** Thanda Aung, Mia Celestin

**Affiliations:** 1 Rheumatology, University of California Los Angeles, David Geffen School of Medicine, Los Angeles, USA

**Keywords:** dactylitis, infectious disease, inflammatory arthritis, mycobacterium abscessus complex, rheumatology

## Abstract

*Mycobacterium abscessus *is an environmental, rapid-growing non-tuberculosis *Mycobacterium* commonly found in soil and water. While typically causing pulmonary infections, it can present with musculoskeletal manifestations that mimic inflammatory arthritis. We present a case of a 73-year-old woman who developed progressive hand tenosynovitis following a gardening injury. The clinical presentation initially suggested seronegative spondyloarthritis, particularly due to the presence of dactylitis. However, synovial biopsy and culture ultimately revealed *M. abscessus *subsp. *abscessus *infection. This case highlights the importance of considering atypical infections in the differential diagnosis of apparent inflammatory arthritis, especially with unilateral involvement and inadequate response to conventional therapy.

## Introduction

Mycobacteria are divided into two major groups for the purpose of diagnosis and treatment: *Mycobacterium tuberculosis* complex, which comprises M. *tuberculosis*, and nontuberculous mycobacteria (NTM), which comprise all of the other mycobacteria species that do not cause tuberculosis [1-2]. *Mycobacterium abscessus* is an increasingly recognized pathogen capable of causing a wide spectrum of clinical manifestations ​[1-2]. NTM such as *M. abscessus* can cause pulmonary disease resembling tuberculosis, skin and soft tissue infections (SSTIs), central nervous system infections, bacteremia, and ocular and other infections ​[1-3]​. The two major mechanisms for acquiring an *M. abscessus* complex-associated SSTI are by 1) direct contact with contaminated material or water through traumatic injury, surgical wound, or environmental exposure and 2) secondary involvement of skin and soft tissue during disseminated disease [2]. The diagnosis of *M. abscessus *infection can be challenging due to its ability to mimic other inflammatory conditions, particularly when presenting as tenosynovitis or dactylitis. This diagnostic challenge is further complicated by the organism’s slow growth and the need for specific culture conditions ​[2]​. Understanding these atypical presentations is crucial for clinicians, as delayed diagnosis can lead to significant morbidity and inappropriate treatment.

## Case presentation

A 73-year-old woman presented to the rheumatology clinic with a one-month history of progressive right thumb pain and swelling. Her symptoms began after pricking her finger while gardening. The patient’s past medical history was significant only for hypertension and hyperlipidemia, with no prior rheumatological conditions. Her family history was negative for autoimmune diseases. She denied constitutional symptoms such as fever, chills, night sweats, or fatigue. She also had no respiratory symptoms, recent gastrointestinal infections, or urinary tract infections that might suggest reactive arthritis. The patient reported no recent travel history. 

**Figure 1:**
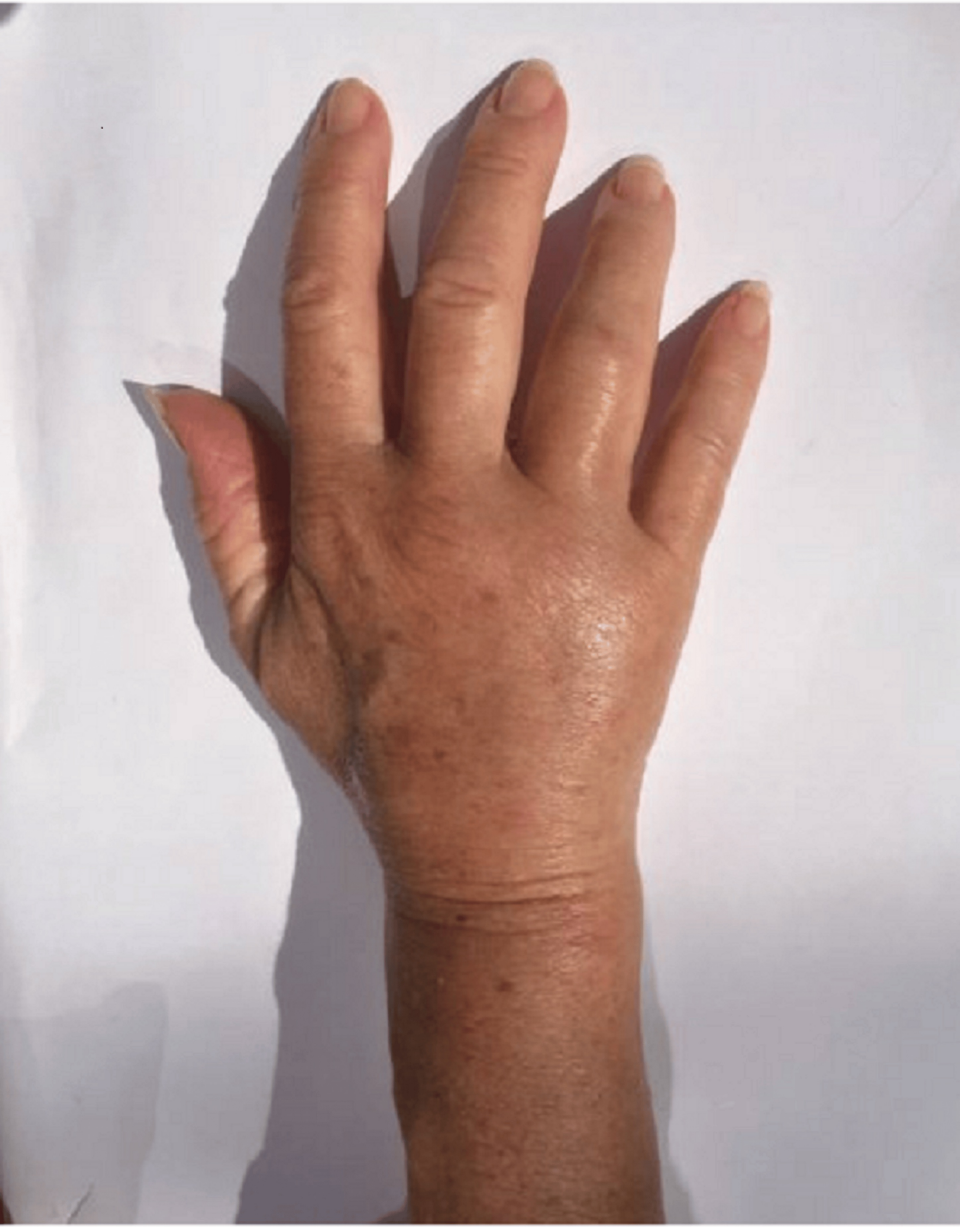
An image of the patient’s right hand at the time of initial visit, with multiple-digit dactylitis

On initial examination, the most striking finding was unilateral dactylitis affecting multiple digits of the right hand, showing "sausage-like" swelling with associated warmth, tenderness, and decreased range of motion both passive and active (Figure 1). No other joints were two months after her initial presentation involved, and the remainder of the physical examination and initial labs were unremarkable (Table 1). 

**Table 1:** Comprehensive metabolic panel and complete blood count

Pertinent lab data	Patient's lab values	Reference range
Sodium (mmol/L)	144	135-146
Potassium (mmol/L)	4.5	3.6-5.3
Chloride (mmol/L)	103	96-106
Total CO_2_ (mmol/L)	28	20-30
Anion gap (mmol/L)	13	8.0-19.0
Glucose (mg/dL)	89	65-99
eGFR (mL/min/1.73m2)	68	60-89
Creatinine mg/dL	0.86	0.60-1.30
Urea nitrogen (mg/dL)	20.0	7.0-22.0
Calcium (mg/dL)	8.9	8.6-10.3
Total protein (g/dL)	6.1	6.1-8.2
Albumin (g/dL)	4	3.9-5.0
Bilirubin, total (mg/dL)	0.3	0.1-1.2
Alkaline phosphatase (U/L)	50	37-113
Aspartate aminotransferase (U/L)	20	13-47
Alanine aminotransferase (U/L)	18	8.0-64.0
White blood cell count (1/uL)	6.35	4.16-9.95 x10E3
Red blood cell count (1/uL)	4.11	3.96-5.09 x10E6
Hemoglobin (g/dL)	11.7	11.6-15.2
Hematocrit (%)	36.5	34.9-45.2
Mean corpuscular volume (fL)	88.8	79.3-98.6
Mean corpuscular hemoglobin (pg)	28.5	26.4-33.4
MCH concentration (g/dL)	32.1	31.5-35.5
Red cell distribution width - SD (fL)	43.7	36.9-48.3
Red cell distribution width - CV (%)	13.3	11.1-15.5
Platelet count, auto (1/uL)	412	143-398 x10E3
Mean platelet volume (fL)	10.8	9.3-13.0
Absolute nucleated RBC count (1/uL)	0	0.00-0.00 x10E3
Neutrophil Abs (1/uL)	4.69	1.80-6.90 x10E3
Absolute lymphocyte count (1/uL)	1.05	1.30-3.40 x10E3
Absolute mono count (1/uL)	0.4	0.20-0.80 x10E3
Absolute Eos count (1/uL)	0.12	0.00-0.50 x10E3
Absolute Baso count (1/uL)	0.04	0.00-0.10 x10E3
Absolute immature granulocyte count (1/uL)	0.05	0.00-0.04 x10E3

Inflammatory markers showed an elevated erythrocyte sedimentation rate (ESR) at 90 mm/hr, although C-reactive protein (CRP) remained normal at less than 0.8 mg/dL. Rheumatologic workup, including rheumatoid factor, anti-CCP antibodies, ANA, anti-SSA, anti-SSB antibodies, and HLA-B27, was negative; her uric acid level was also normal (Table 2). The patient's chest radiograph showed no abnormalities.

**Table 2:** Labs upon presentation to Rheumatology ANA: antinuclear antibody; SSA: Sjögren’s-syndrome-related antigen A; SSB: Sjögren’s-syndrome-related antigen B; Cyclic Citrulline Ab IgG: cyclic citrulline antibody immunoglobulin G; ESR: erythrocyte sedimentation rate, HLA: human leukocyte antigen;  MTB-Quantiferon-Gold ELISA: *Mycobacterium tuberculosis*-Quantiferon-GOLD enzyme-linked immunosorbent assay

Pertinent lab data	Patient's lab values	Reference range
ANA Ab titer	<1:40	<1:40
SSA (U)	<20	<20
SSB (U)	<20	<20
Rheumatoid factor (IU/mL)	< 10	<25
Cyclic citrulline Ab IgG (units)	8	0 - 19
Cryocrit	Negative	Negative
ESR (mm/hr.)	90	<=12
C-reactive protein (mg/dL)	0.7	<0.8
Uric acid (mg/dL)	4.7	2.9 - 7.0
HLA B 27	Not present	Present or Not Present
MTB-Quantiferon-Gold ELISA	Positive	Positive or not positive

**Figure 2:**
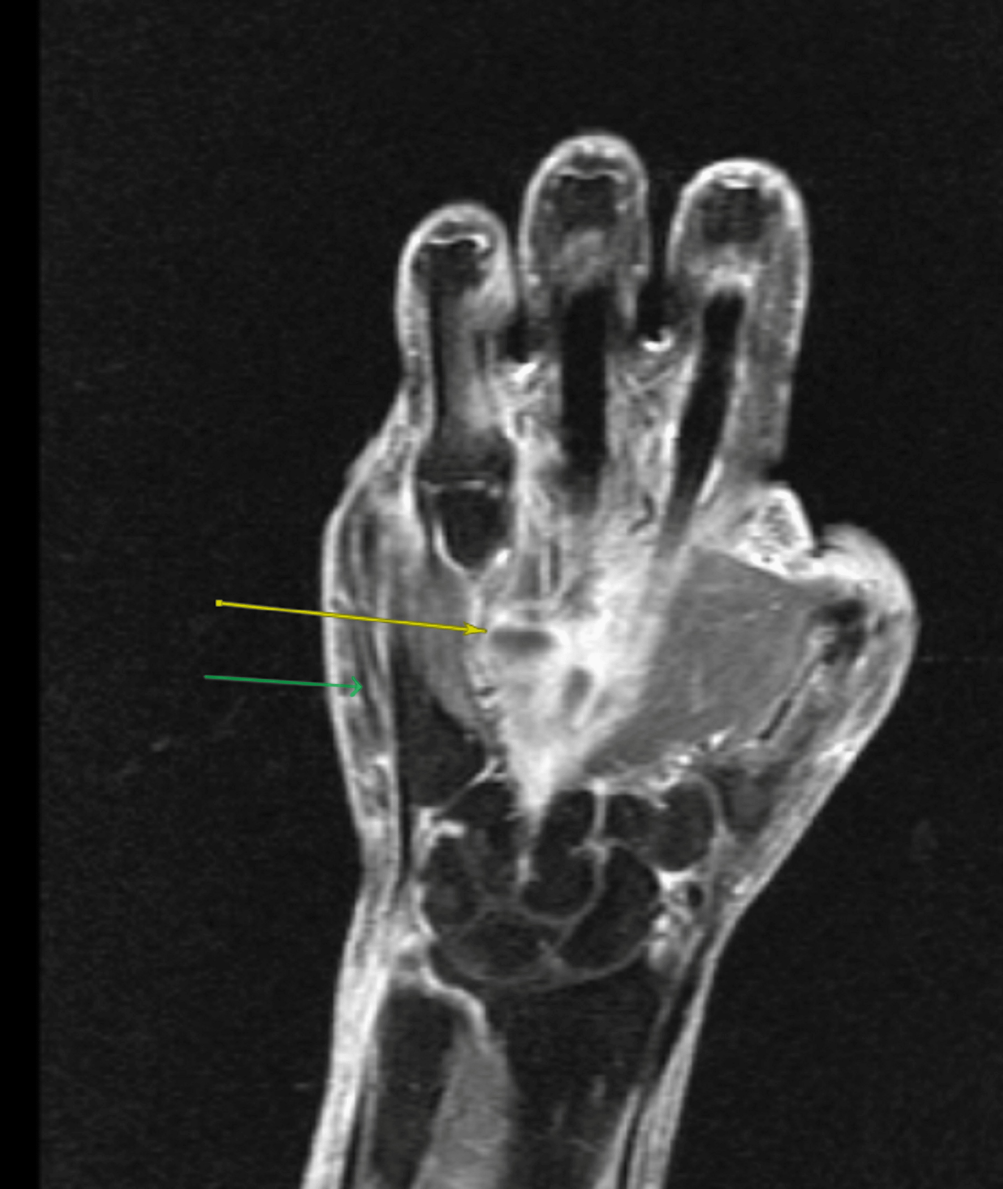
MRI of the right hand showing tenosynovitis from the distal forearm to the palm (yellow arrow), with diffuse subcutaneous edema (green arrow).

The patient was initially treated for presumed cellulitis with cephalexin without significant improvement. Inflammatory arthritis such as seronegative rheumatoid arthritis or dactylitis secondary to spondyloarthropathy was considered in the differential diagnosis. Given the unilateral presentation and negative rheumatologic workup, seronegative rheumatoid arthritis was considered unlikely. Although HLA-B27 was negative, spondyloarthropathy remained a possible diagnosis. Due to persistent severe symptoms, the patient was subsequently treated with oral methylprednisolone (six-day Medrol pack) and non-steroidal anti-inflammatory drug, ibuprofen 400 mg PO every eight hours as needed. While these interventions provided temporary relief, her symptoms recurred with greater severity. The inflammation progressively spread to involve other digits and the palm of her right hand. The MRI of the right hand demonstrated synovitis of multiple digits without erosive changes or osteomyelitis (Figure 2). 

Two months after her initial presentation, the clinical course took a significant turn when the patient presented to the emergency department with severe right-hand pain and swelling without neurological deficits. Despite initial treatment with ampicillin-sulbactam, her symptoms persisted. Due to severe pain and functional limitation and limited range of movement of the fourth digit both actively and passively, she underwent surgical exploration with flexor tendon sheath release and debridement. Intraoperative cultures ultimately grew Mycobacterium *abscessus* subsp. *abscessus. *

**Figure 3:**
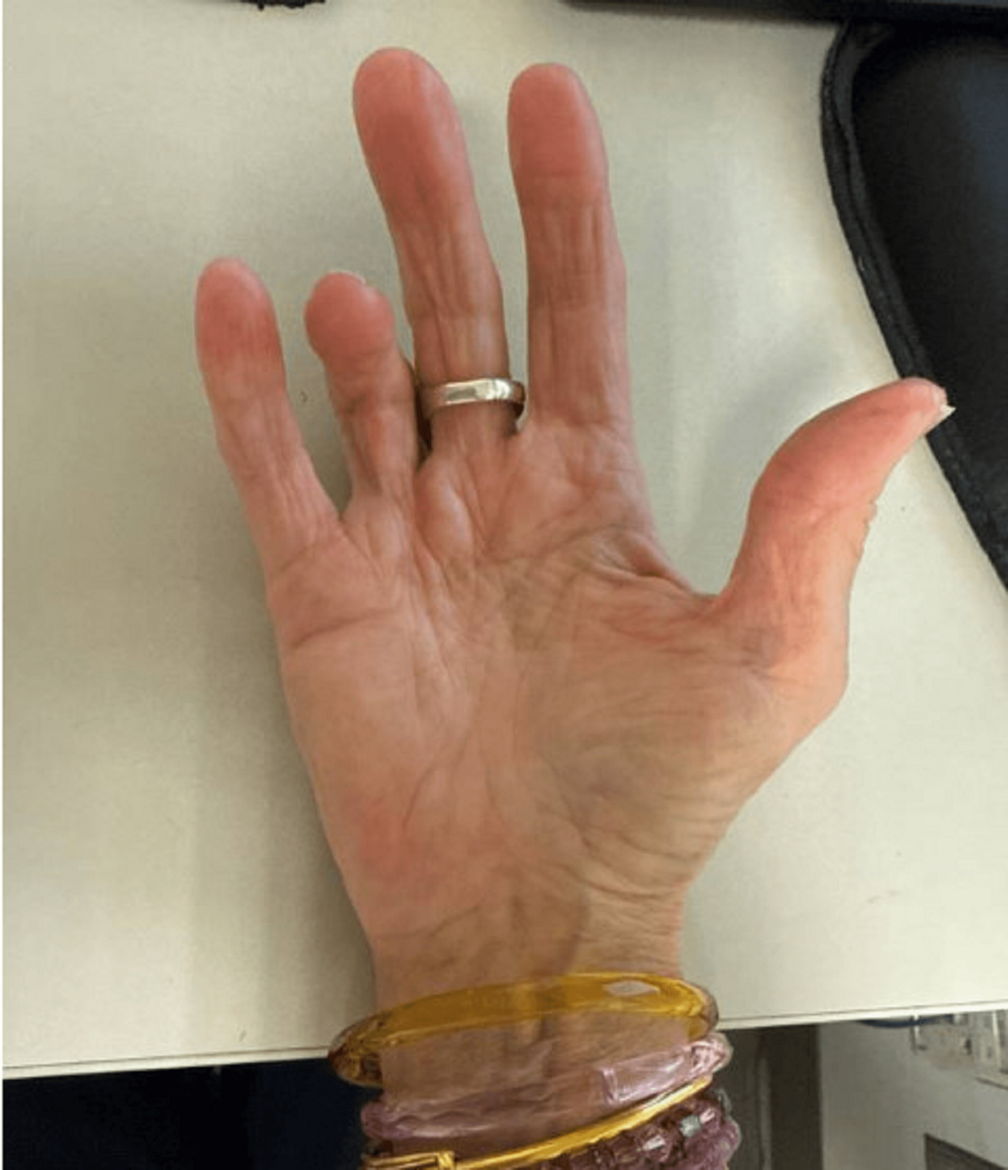
An image of the patient’s right hand seven years after surgical debridement

Following identification of the pathogen, the patient was initiated on combination intravenous therapy with amikacin for four months, linezolid for two months, tedizolid for four months, and imipenem for six months; antibiotic selection was based on susceptibility testing. This resulted in complete resolution of the infection with only minimal residual tenderness, and her sedimentation rate also normalized. No recurrence was noted during follow-up. However, there was a residual contracture affecting the fourth digit seen at her follow-up seven years later (Figure 3).

## Discussion

This case illustrates several important clinical considerations in diagnosing and managing atypical mycobacterial infections. The presentation of M.* abscessus* infection as unilateral dactylitis posed a significant diagnostic challenge, as these findings typically suggest seronegative spondyloarthritis or reactive arthritis ​[4]​. The diagnostic complexity was compounded by multiple factors that obscured the underlying infectious etiology. The absence of systemic symptoms typical of infection initially steered clinical suspicion away from an infectious process. Furthermore, the temporary response to corticosteroids masked the underlying infection and might have also worsened the infection since corticosteroids suppresses the immune system. Normal rheumatology serologies such as rheumatoid factor, CCP, and HLA B27 negativity added another layer of diagnostic uncertainty, while the clinical features closely mimicked those typically seen in inflammatory arthritis ​[5]​. 

The case provides valuable insights for clinical practice, particularly regarding the approach to unilateral joint involvement. When encountering unilateral joint manifestations, clinicians should maintain a high index of suspicion for infection or trauma rather than systemic inflammatory disease which often present with bilateral joints manifestation. This becomes especially relevant in cases with a clear history of environmental exposure or minor trauma, as seen in our patient's gardening injury ​[6]​. Environmental mycobacteria deserve consideration in cases of persistent soft tissue infection following exposure to soil or water, even in immunocompetent hosts ​[2-3]​. 

The role of surgical intervention proved crucial in this case, both for diagnostic and therapeutic purposes. Surgical debridement provided tissue for definitive diagnosis and facilitated infection control ​[7]​. This highlights the importance of considering early surgical evaluation in cases of persistent, unexplained joint inflammation, particularly when standard therapeutic approaches fail to provide lasting improvement. The successful outcome in this case was achieved through a combination of surgical debridement and 6-months of antimicrobial therapy, underscoring the necessity of a comprehensive treatment approach for mycobacterial infections. 

The increasing recognition of *M. abscessus *as a pathogen in immunocompetent hosts represents an evolving understanding of its clinical significance. While traditionally associated with pulmonary disease in immunocompromised patients, this case demonstrates its capacity to cause localized infection following minor trauma ​[1,6,8]​. This changing epidemiology emphasizes the need for clinicians to remain vigilant for atypical presentations of mycobacterial infections, even in otherwise healthy individuals. 

Furthermore, this case demonstrates the value of maintaining a broad differential diagnosis and avoiding premature diagnostic closure. The initial presentation's similarity to inflammatory arthritis could have led to continued inappropriate treatment with immunosuppressive agents, potentially worsening the infection. Instead, the persistence in pursuing a definitive diagnosis through surgical biopsy and culture ultimately led to appropriate treatment and favorable outcomes ​[9]​. 

## Conclusions

This case highlights the importance of maintaining a broad differential diagnosis when evaluating patients with apparent inflammatory arthritis, particularly when the presentation is unilateral or atypical. The overlap between infectious and inflammatory conditions necessitates careful clinical assessment and appropriate use of diagnostic testing, including tissue biopsy when indicated. Recognition of atypical presentations of *M. abscessus* infection is crucial for timely diagnosis and appropriate management. This case also underscores the value of surgical debridement in both establishing the diagnosis and facilitating the successful treatment of mycobacterial tenosynovitis.
